# A β-Thalassemia Cell Biobank: Updates, Further Validation in Genetic and Therapeutic Research and Opportunities During (and After) the COVID-19 Pandemic

**DOI:** 10.3390/jcm14010289

**Published:** 2025-01-06

**Authors:** Roberto Gambari, Maria Rita Gamberini, Lucia Carmela Cosenza, Cristina Zuccato, Alessia Finotti

**Affiliations:** 1 Center “Chiara Gemmo and Elio Zago” for the Research on Thalassemia, Ferrara University, 44121 Ferrara, Italy; gamberinimariarita@gmail.com; 2 Department of Life Sciences and Biotechnology, Ferrara University, 44121 Ferrara, Italy; luciacarmela.cosenza@unife.it (L.C.C.); cristina.zuccato@unife.it (C.Z.)

**Keywords:** biobanking, beta-thalassemia, erythroid precursor cells, fetal hemoglobin, gene editing, COVID-19 pandemic

## Abstract

**Background**: Cellular biobanks are of great interest for performing studies finalized in the development of personalized approaches for genetic diseases, including β-thalassemia and sickle cell disease (SCD), important diseases affecting the hematopoietic system. These inherited genetic diseases are characterized by a global distribution and the need for intensive health care. The aim of this report is to present an update on the composition of a cellular Thal-Biobank, to describe its utilization since 2016, to present data on its application in studies on fetal hemoglobin induction and on gene editing, and to discuss its employment as a “unique tool” during and after the COVID-19 pandemic. **Methods**: The methods were as follows: freezing, cryopreservation, long-term storage, and thawing of erythroid precursor cells from β-thalassemia patients; fetal hemoglobin (HbF) induction; CRISPR-Cas9 gene editing; HPLC analysis of the hemoglobin pattern. **Results**: The updated version of the Thal-Biobank is a cellular repository constituted of 990 cryovials from 221 β-thalassemia patients; the phenotype (pattern of hemoglobin production) is maintained after long-term storage; fetal hemoglobin induction and CRISPR-Cas9 gene editing can be performed using biobanked cells. In representative experiments using an isoxazole derivative as HbF inducer, the HbF increased from 13.36% to more than 60%. Furthermore, in CRIPR/Cas9 gene editing, de novo production of HbA was obtained (42.7% with respect to the trace amounts found in untreated cells). **Conclusions**: The implemented Thal-Biobank was developed before the COVID-19 outbreak and should be considered a tool of great interest for researchers working on β-thalassemia, with the aim of developing innovative therapeutic protocols and verifying the impact of the COVID-19 pandemic on erythroid precursor cells.

## 1. Introduction

Biobanking of biological material and construction of biorepositories are activities significantly expanding their applications in biomedicine and applied biotechnology. They are important in the fields of data sharing, shared use of precious biomaterials, and development of commonly designed and validated protocols in molecular and cellular biology [1,2,3,4]. The term “biobank” is rather general, including biological samples such as plasma, serum, whole cells and tissues, urine, buffy coats, RNA, genomic DNA, and protein extracts [5,6]. An increasing number of diverse biobanks are present worldwide, as discussed by O’Donoghue et al. [7], many of them of great interest to health researchers [8,9]. In this respect, the quality of the biobank, the updated description of its features, and access to the biological material are all crucial parameters for the possible use of the biobank in biomedicine [9,10]. In relation to these parameters, with particular reference to the availability of biological materials, the type of adopted governance strategies is a crucial step in facilitating access to biobanked material (including datasets) by the research community [11]. 

In this respect, one of the most appealing projects is to construct “cellular biobanks” [12,13,14,15,16] for possible future use in the post-genomic era [3]. The reason for this interest is that “cellular biobanks” are expected to be very important in developing personalized medicines [4,12,17], providing “ready-to-use” biomaterials for developing (a) therapeutic-oriented protocols based on the use of bio-drugs (low-molecular-weight compounds, microRNAs, antisense oligonucleotides, others), (b) products for gene therapy (viral and non-viral), and (c) products for gene editing. Biobanking should be considered an issue of global interest [8,18], and it is a growing field of research and biomedical applications in low- and middle-income countries (LMICs), forcing the scientific community to discuss novel legal and policy issues related to this key activity [19,20]. It can be concluded that serious attention should be given to the activity and perspectives of researchers working on biobanking-related investigations in LMICs [19]. Data sharing and exchanges of unique biobanked samples are issues of great importance to guarantee the global usability of the products and results obtained in this sector of applied research.

A biomedical area of research that needs validated biobanked collections is related to genetic diseases [21], including β-thalassemia and sickle cell disease (SCD), important diseases affecting the hematopoietic system [22,23,24]; notably, these inherited genetic diseases are characterized by a global distribution and the need for intensive health care and research efforts [25,26], with the objective of developing cost-effective treatments [27,28,29].

In this context, we produced and validated a cell biobank for β-thalassemia [30], noting that the use of cells from the biobank allows for the stratification of patients with respect to the endogenous production of fetal hemoglobin and can determine the response to treatment with fetal hemoglobin inducers and gene therapy protocols with reproducible results. In particular, we were able to demonstrate that the use of biobanked samples allows for the simultaneous comparison of many potentially therapeutic treatments across several samples grown in parallel. Furthermore, in the study reported by Cosenza et al. [30], the biobanked samples were validated for retention of the phenotype in relation to hemoglobin production after thawing frozen cells isolated from the same patient at different times. In addition, different frozen batches of the same patient’s cells retained the same hemoglobin pattern. This reproducibility was also found in cultures of the same biobanked cells that were sent to different laboratories [30]. Finally, the biobanked cells allow for stratification of the patients with respect to fetal hemoglobin (HbF) production and can be used for determining the response to HbF inducers, as discussed in a recent review article [31]. Recent reviews on HbF inducers in β-thalassemia are also available [32,33,34,35,36]. 

The collection of cellular samples for thalassemia research is a key issue of several EU-funded projects, such as the Thalassemia Modular Stratification System (THALAMOSS) for personalized therapy of beta-thalassemia [31]. Furthermore, this field of investigation is central in two ongoing projects related to hemoglobinopathies: the COST-action CA22119–HELIOS [37] and the International Hemoglobinopathy Research Network (INHERENT) project [38], which brings together nine existing international or regional consortia in the field of hemoglobinopathies, namely ITHANET, RADeep, ARISE, SPARCO, SADaCC, REDAC, the HVP Global Globin Network, the International Health Repository, and the ClinGen Hemoglobinopathy VCEP, as reported by Gambari et al. [31].

The Thal-Biobank reported by Cosenza et al. [30] was a cellular repository for research on thalassemia and sickle cell disease that was composed of 779 biobanked specimens from 8 healthy donors and 72 patients with sickle cell disease (SCD) and β-thalassemia. The most important difficulty of the study was related to the recruitment of patients. In fact, the distribution of the genotypes was not uniform, the most frequent genotypes being β^0^39/β^0^39 (29 patients), β^+^IVSI-110/β^0^39 (17 patients), and β^+^IVSI-110/β^+^IVSI-110 (8 patients). On the contrary, a limited number of patients with the β^+^IVSI-110/β+IVSI-6 and β^+^IVSI-110/β+IVSI-1 genotypes were recruited. Few patients with at least one SCD allele were recruited, and their erythroid cells biobanked (7 patients, 52 vials). As far as the validation of protocols for their impact in the therapeutic field of β-thalassemia is concerned, no attempt was made to determine the phenotypic correction of biobanked erythroid cells using the gene-editing approach.

The objectives of the present report were (a) to present updates on the composition of the cell Thal-Biobank, (b) to describe its utilization since 2016, (c) to present novel data on the application of biobanked cells in studies on fetal hemoglobin induction and gene editing, and (d) to discuss the utilization of the Thal-Biobank as a “unique tool” during and after the COVID-19 pandemic, taking in consideration the fact that all the patients participating to this study were not SARS-CoV-2-infected and COVID-19-vaccinated. Therefore, the described Thal-Biobank should be considered composed of “SARS-CoV-2-free” cells to serve as precious reference control of SARS-CoV-2-infected erythroid cells in studies aimed at characterizing the effects of SARS-CoV-2 infection and COVID-19 vaccination on the hematopoietic system. 

In this respect, recent reports concurrently demonstrated that (a) a perturbation of the hematopoietic system occurs in COVID-19 patients [39,40,41,42,43,44], (b) erythroid precursors can be infected by SARS-CoV-2 [45,46], and (c) infection of SARS-CoV-2 is associated with deep alteration of erythroid differentiation properties [47,48] and transcriptomic profile [49,50,51]. In particular, the multi-omics study by Bernardes et al. [49] provides strong evidence of broad cellular effects of SARS-CoV-2 infection beyond adaptive immune cells with deep involvement of the erythroid compartment. Notably, the cell Thal-Biobank described by Cosenza et al. [30] (and its implementation described in this report) was produced before the global COVID-19 pandemic outbreak. Therefore, it could represent an excellent reference for verifying possible phenotypic alterations induced by the infection by SARS-CoV-2 and by the COVID-19 vaccination in individual patients who were donors of biobanked cells and subsequently infected with SARS-CoV-2 and/or vaccinated against COVID-19.

## 2. Materials and Methods

### 2.1. Patients

Patients were recruited following all the ethical requirements and the approval of the Ethical Committees of Ferrara Hospital and Rovigo Hospital. The blood samples were collected from β-thalassemia and sickle-cell disease (SCD) patients after the informed consent form was signed. 

### 2.2. Isolation and Culture of Peripheral Blood Cells

Peripheral blood mononuclear cells (PBMCs) (about 25 mL) were collected in Vacutainer LH-treated tubes (BD Vacutainers, Becton Dickinson, Berkshire, UK). PBMC isolation was obtained from whole blood by Ficoll–Hypaque density gradient centrifugation (Lympholyte^®^-H Cell Separation Media, Cedarlane, Euroclone, Italy). After the separation of the various blood components, the ring was harvested and washed once with 1X Dulbecco’s Phosphate Buffered Saline without Ca and Mg (DPBS W/O CA-MG, GIBCO, Invitrogen, Life Technologies, Carlsbad, CA, USA). CD34^+^ cells were selected from PBMCs using anti-CD34^+^ magnetic microbeads and magnetic-activated cell sorting separation LS columns (both from Miltenyi Biotec, Bergisch Gladbach, Germany) according to the manufacturer’s protocol. The separation in the column was performed twice to increase the CD34^+^ cells’ purification. The obtained cells were maintained in culture with two different protocols, one to obtain a differentiation phenotype (Protocol A), the other for cellular expansion before the freezing procedure (Protocol C). Protocols A and C are described in Supplementary Methods and were originally described by Cosenza et al. [30]. 

### 2.3. Freezing, Cryopreservation, and Thawing

Once achieved the maximum expansion using the Protocol C, CD34^+^ cells were frozen following the Naldini’s method: Iscove’s modified Dulbecco’s Medium (IMDM, Life Technologies, Carlsbad, CA, USA) with 50% FBS (Celbio, Milan, Italy) and 10% Dimethyl Sulfoxide RPE-ACS (DMSO, Carlo Erba, Italy). The cells were immediately placed at −80 °C and subsequently stored in liquid nitrogen (−147 °C). Each vial present in the cellular Thal-Biobank contains 1–5 × 10^6^ cells. The cells were thawed in 5 mL of IMDM (Life Technologies, Carlsbad, CA, USA), 5% FBS, and incubated at 37 °C with controlled humidity with 5% CO_2_ in an expansion medium compound as described earlier.

### 2.4. Quality Control Procedures

Quality control is required to ensure possible applications of biobanked cells to biomedicine [52,53,54,55]. The infrastructures and procedures of the biobank quality control system are the following: (a) the cryo room is equipped with a liquid nitrogen refrigerator dedicated to the storage of the biobanked cryovials; (b) the access to this equipment is restricted to researchers involved in the biobank management; (c) freezers and CO_2_ incubators are equipped with alarm systems to check temperature and CO_2_ variations; (d) in our cell culture laboratory two CO_2_ incubators are dedicated to experiments concerning the “biobank project”; (e) routine mycoplasma contamination assessment in cell cultures is performed, before the freezing and after the thawing procedures, using the Venor**^®^**GeM Classic Mycoplasma Detection Kit (cat No. 11-1050, Minerva Biolabs GmbH, Schkopauer Ring 13, D-12681 Berlin, Germany); (f) after thawing, the toxicity is routinely assayed by the Thiazolyl Blue Tetrazolium Bromide assay (cat No. M2128, Sigma-Aldrich, Saint Louis, MO, USA); (g) after thawing, induction of a fraction of the cellular sample with the reference HbF inducer mithramycin (30 nM) is routinely performed. 

### 2.5. Treatment of Long-Storage Biobanked Cells with HbF Inducers 

About four days after thawing, the CD34^+^ cells were treated with HbF inducers. We used 2–3 × 10^6^ cells for each experiment. We tested the cells with different types of HbF inducers, such as mithramycin, hydroxyurea, resveratrol, butyric acid, rapamycin [31,32,33,34,35,36], and isoxazole derivatives [56]. Three independent cell cultures were routinely performed in the presence of EPO and HbF inducers. Controls were constituted by EPO-cultured untreated cells. At the end of the 7-day treatment, the cells were washed twice with 1X DPBS (GIBCO, Invitrogen, Life Technologies, Carlsbad, CA, USA), and from the pellet obtained, analysis of hemoglobin production was performed by HPLC (high-performance chromatography) [57,58] as described elsewhere [59]. 

### 2.6. Treatment of Cells with the β^0^39 CRISPR-Cas9 System

On the third day of phase II, the ErPCs were considered ready for treatment with the β^0^39 CRISPR-Cas9 system. The CRISPR-Cas9 System was employed for correcting the β^0^39-globin gene mutation. Regarding the gene-editing procedure, the protocol described by Cosenza et al. was followed [60]. The genomic sgRNA target sequence was 5′-TGG TCT ACC CTT GGA CCT AGA GG-3′ [60]; the gRNA complex began by joining a tracrRNA (ATTO 550 labeled Alt-R^®^ CRISPR-Cas9 tracrRNA, IDT, Newark, NJ, USA) and the Alt-R^®^ CRISPR-Cas9 crRNA (IDT) oligonucleotide in thermo-block at 95 °C for 5 min. In order to verify gene correction at the RNA level, the relative content of α-, β-, and γ-globin mRNAs was quantified by multiplex qPCR using primers and FAM, HEX, and Cy5/ZEN/IBFQ-labeled hydrolysis probes purchased as custom-designed PrimeTime qPCR Assays from IDT. Droplet Digital PCR (ddPCR) was employed to evaluate the efficiency of the correction of the β-globin gene at the codon 39 position [60,61]. 

## 3. Results

### 3.1. Characterization and Validation of the β-Thal Cell Biobank: Biobanked Samples of the Same β-Thalassemia Patient Maintain the Hemoglobin Pattern After Long-Time (6 Years) Storage

The composition of the first release of the β-Thal Cell Biobank has been reported by Cosenza et al. [30]. It was composed of 779 biobanked cellular pellets from 8 healthy donors and 72 thalassemia and sickle-cell disease (SCD) patients; the majority of recruited patients were β-thalassemia subjects with a β^0^39/β^0^39 genotype.

**Figure 1:**
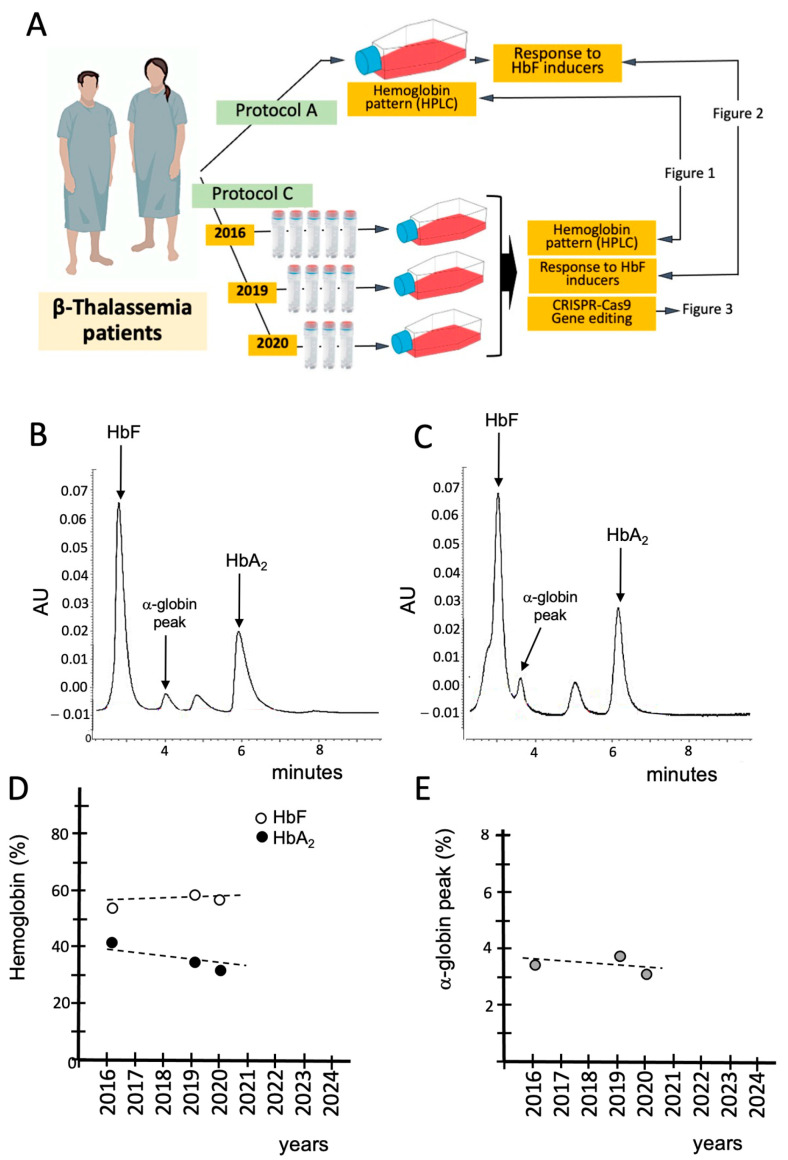
Biobanked samples of the same β-thalassemia patient maintain the hemoglobin pattern after long-term storage. (**A**) The experimental plan of the present study. Results concerning response to HbF inducers are presented in Figure 2; results concerning CRISPR-Cas9 gene editing are presented in Figure 3. (**B**–**E**) Pattern of hemoglobin production when cryo-preserved cells were thawed and sub-cultured in 2016 (**A**) and in 2020 (**B**). (**D**,**E**) Percentage of HbF (**D**, white circles), HbA_2_ (**D**, black circles), and percentage of the α-globin peak (**E**, grey circles) in cells of different vials from the same patient, thawed and sub-cultured in EPO in 2016, 2019, and 2020, as indicated.

In agreement with the experiments proposed by Cosenza et al. [30], an important validation of the β-Thal Cell Biobank was the demonstration that biobanked samples of the same β-thalassemia patients maintain the hemoglobin pattern after long-term storage (see Figure 1). To this end, the HPLC pattern of biobanked cells (after thawing, expansion, and treatment with EPO as described in Section 2.5 of the Materials and Methods section) has been compared with the HPLC pattern obtained from cell cultures of biobanked cells performed before the freezing procedure. 

We had the chance to compare hemoglobin patterns of different vials from the same patient (patient “A”) after a long-time (6 years) storage (from 2016 to 2022) (see the workflow reported in Figure 1A). A representative example is shown in Figure 1B,C depicting the HPLC analysis of the hemoglobin produced by cryopreserved CD34^+^ ErPCs from the same patient, thawed and sub-cultured in the presence of erythropoietin in 2016 (B) and in 2020 (C).

Within the “β-Thal-biobanking project”, we were interested in determining whether the peak referring to fetal hemoglobin (HbF) and the α-globin chain peak were stable over time. We, therefore, compared EPO-cultured cells from a vial of patient “A” taken from the β-Thal Cell Biobank in 2016 with EPO-cultured cells from a second vial of the same patient taken from the same Thal-Biobank in 2019 and 2020 (see the scheme of the experiment shown in Figure 1A).

The HPLC analyses shown in Figure 1B,C demonstrate that the HPLC patterns obtained are very similar when cells from the same patient were sub-cultured in 2016 and in 2020. Panels D and E of Figure 1 show the summary of the hemoglobin pattern by cells biobanked in 2016, 2019, and 2020. As is evident, the % of HbF (Figure 1D, white symbols), HbA2 (Figure 1D, black symbols), and the % of the α-globin peak (Figure 1E, grey symbols) are quite constant, confirming that the phenotype of cryo-preserved ErPCs does not change over time, even in the case of a long period of time. The novelty of this experiment with respect to similar experiments reported by Cosenza et al. [30] is that the storage period of time in the present study was much longer (6 years compared to 8 months). 

### 3.2. Validation of the β-Thal Cell Biobank: Use of Biobanked Samples in Pre-Clinical Studies

Since 2016, the biobank has been employed in several research projects as summarized in Table 1. In total, 428 vials were used (from 106 patients) to carry out experiments related to four projects: (a) induction of HbF with low-molecular-weight compounds (a Task of the Thalamoss Project) [31], (b) induction of HbA with readthrough molecules, (c) genome editing to correct the β^0^39 mutation, and (d) effects of the SARS-CoV-2 Spike protein on hemoglobin production by ErPCs. The results obtained are listed in Table 1. Representative examples of results obtained using materials from the β-Thal Cell Biobank are presented in Section 3.3 and Section 3.4.

### 3.3. Validation of the β-Thal Cell Biobank: An Update on the Induction of Fetal Hemoglobin (HbF) Using Biobanked Samples

**Figure 2:**
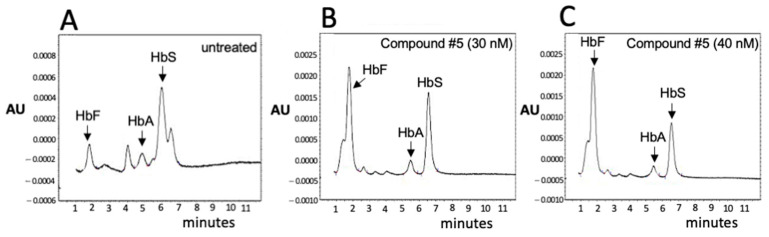
Compound #5 induces increased production of HbF in biobanked ErPCs from a β^0^39/HbS patient. After thawing, EPO-cultured cells were either untreated (**A**) or treated for 5 days with 30 nM (**B**) and 40 nM (**C**) isoxazole derivative #5 [56].

About four days after thawing, the CD34^+^ cells purchased from the β-Thal-Biobank were treated with HbF inducers. In the representative experiment shown in Figure 2, the isoxazole derivative #5 was employed [56]. This was one of the most effective isoxazoles described by Zuccato et al. [56]. For verifying the possibility of studying HbF induction, the cellular pellets obtained after 5 days of induction were used for cytoplasmic extract production for HPLC. The results obtained indicated that the % of HbF increased from 13.36% (in untreated cells, as shown in Figure 2A) to 60.92 and 69.27% in cells treated with 30 (Figure 2B) and 40 (Figure 2C) nM compound #5, respectively.

### 3.4. Validation of the β-Thal Cell Biobank: CRISPR-Cas9-Based Gene Editing Using Biobanked Samples

**Figure 3:**
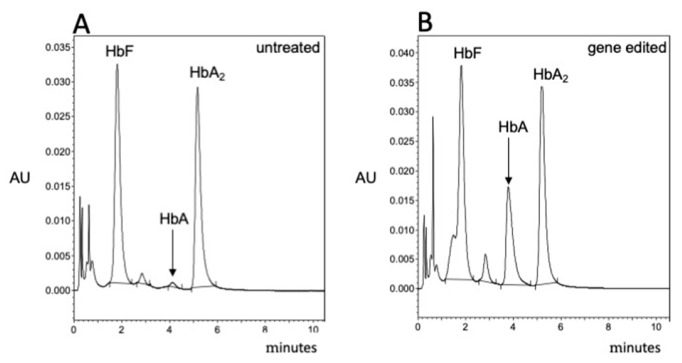
CRISPR-Cas9-based gene editing of cryopreserved cells of a β^0^39/β^0^39 patient. Thawed cells isolated from the β-Thal Cell Biobank were cultured for 5 days and then were untreated (**A**) or gene-edited according to Cosenza et al. [60] using the CRISPR-Cas9 system with forward donor DNA template (**B**). De novo production of HbA in gene-edited cells from the β^0^39/β^0^39 patient is appreciable.

In the context of the development of personalized therapy and gene editing, the most important validation of the β-Thal Cell Biobank is the demonstration that efficient CRISPR-Cas9-based gene editing can be achieved using biobanked CD34^+^ ErPCs. An example of the results obtained with this approach is shown in Figure 3, which reports the HPLC pattern of untreated cells (Figure 3A) or cells edited as described by Cosenza et al. [60] (Figure 3B) using the CRISPR-Cas9 system with a forward donor DNA template.

The results of the experiment performed (Figure 3) clearly show that gene editing is possible after 4 years of storage of biobanked CD34^+^ cells of β^0^39/β^0^39 patients. At present, biobanked CD34^+^ cells from 5 β^0^39/β^0^39 have been thawed, gene-edited, and frozen again to be stored in the biobank as experimentally validated “gene-edited biobanked samples”. 

### 3.5. Updates on the Composition of the Cell β-Thal Cell Biobank

Originally, the composition of the β-Thal Cell Biobank was as follows: 779 biobanked cellular pellets from 8 healthy donors and 72 thalassemia and sickle-cell disease (SCD) patients, the majority being β-thalassemia patients. Since 2016, the cellular Thal-Biobank has been implemented, and it is now composed as reported in Table 2.

The distribution of the recruited patients is, at present, still uneven, as some genotypes are frequent, such as β^0^39/β^0^39 (103 patients), β^+^IVSI-110/β^0^39 (25 patients), and β^+^IVSI-110/β^+^IVSI-110 (9 patients), while other genotypes are rare (such as β+IVSI-110/β+IVSI-6 and β+IVSI-110/β+IVSI-1). Few sickle-cell disease patients were recruited, and their erythroid cells biobanked (10 patients, 57 vials). Regarding SARS-CoV-2 infection and COVID-19 vaccination, the analysis for future recruitment is still ongoing. During a first screening, among 74 patients from whom the erythroid cells included in the biobank were isolated, 8 were infected with SARS-CoV-2 and 30 underwent anti-COVID-19 vaccination (or with Pfizer’s BNT162b2 vaccine, or with Moderna’s mRNA-1273).

## 4. Discussion

The availability of biobanked cells for research on β-thalassemia is of great interest in order to perform large-scale experiments avoiding recruitment of patients for the necessary biological samples. In the case of biobanked samples, a new recruitment is not required, as we can take biobanked cells, without restrictions concerning the number of samples, and the genotype, or the basal level of fetal hemoglobin. The only issue to be considered with great attention is the quality of collected biospecimens and materials, which is crucial in the case of use by different research groups [5,6,52,53,54,55]. To ensure the highest level of quality, great care is required when handling blood sample collection, isolation of erythroid precursor cells, freezing, and long-term storage.

In this study, we report the use (Table 1) of the Thal-Biobank described by Cosenza et al. in 2016 [30] and describe representative examples demonstrating that biobanked cells can be employed to screen and characterize inducers of fetal hemoglobin (see Figure 2) and to validate protocols for gene editing (Figure 3). An important finding supporting the quality of our cellular biobank is that long-term storage (6 years) did not alter the pattern of hemoglobin production (characterized by HPLC analysis) when biobanked CD34^+^ cells were isolated, thawed, and sub-cultured in the presence of erythropoietin (EPO).

In this paper, we also present the updated composition of the Thal-Biobank. The comparison of the number of biobanked cells with the original Biobank described by Cosenza et al. [30] indicates an increased number of biobanked cryovials from 779 (from 80 subjects) to 990 (from 221 subjects). At the same time, a total of 428 biobanked cryovials have been used in projects related to β-thalassemia, as outlined in Table 1.

The first conclusion of our study is that the implemented Thal-Biobank should be considered a useful tool to sustain research on β-thalassemia, of potential interest for developing innovative therapeutic protocols.

A final but, at least in our opinion, very important comment is based on the fact that the biobanked cells were isolated from β-thalassemia patients before the COVID-19 pandemic outbreak [64,65,66]. Accordingly, all the patients recruited for the isolation of erythroid cells to be biobanked did not exhibit symptoms related to COVID-19. After the COVID-19 pandemic outbreak, some of them have been infected by SARS-CoV-2, and most of them have been vaccinated with anti-SARS-CoV-2 vaccines (or with Pfizer’s BNT162b2 vaccine, or with Moderna’s mRNA-1273) [67,68].

In this regard, it should be underlined that COVID-19 and the associated SARS-CoV-2 infection might have important effects on erythropoiesis. The possible impact of SARS-CoV-2 infection and COVID-19 vaccination on the hematopoietic system has been confirmed by several studies [39,40,41,42,43,44,69,70,71,72]. For instance, Ropa et al. found that SARS-CoV-2 S protein inhibits the expansion of hematopoietic stem/progenitor cells and deeply alters the colony-forming capacity. These effects were obtained by a simple exposure to S protein, even in the absence of a true SARS-CoV-2 infection [70]. The impactful results obtained by Estep et al. indicated that SARS-CoV-2 infection and COVID-19 vaccination impair the functionalities and survival of HSPCs in the umbilical cord blood [72]. In our own laboratory, a study has been published suggesting that the BNT162b2 vaccine inhibits globin gene expression when administered to erythroid cells [63]. One of the hypotheses is that the SARS-CoV-2 Spike protein might exhibit “adverse” effects on hematopoietic cells, as suggested by Trougakos et al. [73]. Since infected erythroid cells may present an altered transcriptomic and/or proteomic profile, transcriptomic and proteomic studies, as recently published [49,50,51], are crucial in order to fully understand the impact of SARS-CoV-2 infection, the Spike protein, and COVID-19 vaccination on erythroid precursor cells. 

In this context, we have the unique opportunity (a) to generate post-COVID-19 biobanks consisting of erythroid cells isolated from β-thalassemia patients infected with SARS-CoV-2 and/or vaccinated against COVID-19 and (b) to compare these erythroid cells with those present within the “SARS-CoV-2 free (pre-COVID-19)” Thal-Biobank described and characterized by Cosenza et al. [30] and in this study. It should be noted that many patients who had donated the erythroid cells present within the Thal-Biobank were subsequently infected with SARS-CoV-2 (some of them developing severe forms of COVID-19) and most of them were subsequently vaccinated against COVID-19. We therefore envisage the production of other cellular biobanks containing erythroid precursors from patients who donated the cells for the pre-COVID-19 Thal-Biobank and were later infected by SARS-COV-2 or vaccinated against COVID-19. The comparison of the phenotypes of the erythroid cells obtained from these patients with those of cells of the same patients currently biobanked in our Thal-Biobank might be very helpful in the understanding person-to-person variation in the effects on erythroid cells of COVID-19, PC (post-COVID-19), PASC (Post-Acute Sequelae of COVID-19) and COVID-19 vaccination.

As a final comment, we would like to underline that our β-Thal Cell Biobank should be at present considered as a “research cellular repository” to be used for collaborative projects. Few SCD patients have been recruited for our Biobank (10 patients, 57 vials); however, a project on the construction of an SCD Biobank (CureSCi, Cure Sickle Cell Initiative) was activated in 2021-2022 to construct a Sickle Cell Hematopoietic Stem Cell Bank (SCBank) (https://biolincc.nhlbi.nih.gov/studies/curesc_scd/; accessed on 12 December 2024). 

Among future perspectives, further recruitment of patients and experimental validations are necessary. Concerning the recruitment of patients, the objective is to include patients with genotypes that are still absent in the present composition of the Thal-Biobank. Concerning further experimental validation, the objective is to verify the impact of the biobank, focusing on novel applications in the biomedical research field. This research should include an analysis of possible changes in OMICS profiling in relation to the storage conditions. In the case of the positive results described here being confirmed, and depending on the available funds, infrastructures, and personnel, we will consider the possibility of creating a “fully organized” biobank [8,9,10] according to recently published guidelines (https://www.coe.int › biobanks for Europe) (accessed on 9 December 2024) [74], after confirming that (a) all the ethical, legal, and social issues (ELSIs) [75] and (b) all the regulatory and governance-related aspects [11,76] have been fully addressed. An important point will be the need for effective collaboration with external users [9,10]. This will be done in strict collaboration with the associations of thalassemia patients, since this type of intervention should consider implications related to the patients’ rights in medical research (https://www.coe.int/en/web/bioethics/biobanks; accessed on 9 December 2024). In this regard, among the legal issues to consider, we cite some examples, such as updating the “contract” between the biobank and the donors [77,78] and updating the informed consent form. Finally, the possibility of granting the biobank and its users the right to patent scientific findings obtained using the biobank resources should be considered very carefully [79,80].

## 5. Conclusions

In this study, we report evidence supporting the concept that the implemented β-Thal Cell Biobank (see Table 2) should be considered a tool of great interest for researchers working on **β**-thalassemia, with the aim of developing innovative therapeutic protocols. A second conclusion is that the implemented β-Thal Cell Biobank should be considered “SARS-CoV-2 free” and therefore could be taken into consideration for future studies aimed at comparing erythroid cells from SARS-CoV-2-infected patients with erythroid cells from the same patients isolated before the SARS-CoV-2 infection. 

## Figures and Tables

**Table 1:** Use of biobanked samples (vials) to sustain research on Thalassemia.

THALAMOSS Project: Induction of HbF with LMW Drugs		Project: Induction of HbA with Readthrough-Based Molecules		Project: β^0^39 CRISPR-Cas9 Genome Editing System		Project: Effects of SARS-CoV-2 Spike Protein on ErPCs	
Number of patients	Number of Vials	Number of patients	Number of Vials	Number of patients	Number of Vials	Number of patients	Number of Vials
84	379	5	10	11	27	6	12
Publications: Zuccato et al., 2021 [62]; Zuccato et al., 2022 [59]; Zuccato et al., 2023 [56]		Unpublished		Publications: Cosenza et al., 2021 [60]; Cosenza et al., 2022 [61]		Publications: Cosenza et al., 2024 [63]	

**Table 2:** Implementation of the β-Thal Cell Biobank from 2016.

	Cosenza et al., 2016 [26]		Present Composition of the Biobank	
Genotype/Phenotype	Number of Patients	Number of Vials	Number of Patients	Number of Vials
β^0^39/β^0^39	29	260	103	421
β^+^IVSI-110/β^+^IVSI-110	8	81	9	65
β^+^IVSI-110/β^0^39	17	191	25	51
β^+^IVSI-6/β^+^IVSI-6	2	15	3	28
β^+^IVSI-6/β^0^39	4	67	5	24
β^+^IVSI-110/β^+^IVSI-6	1	11	1	8
β^+^IVSI-110/β^+^IVSI-1	1	7	1	7
SCD/SCD	2	13	3	17
SCD/β^0^39	1	7	2	10
SCD/β^+^IVSI-110	1	5	2	8
SCD/β^+^IVSI-6	1	7	1	7
SCD/β^+^IVSI-1	2	20	2	15
Others	11	95	64	329
Total	80	779	221	990

## Data Availability

Data will be made available upon reasonable request to the corresponding authors. The biological material will be made available on the basis of a scientific joint collaboration and after verification that all the ethical and legal issues are met.

## Supplementary Materials

The following supporting information can be downloaded at: https://www.mdpi.com/article/10.3390/jcm14010289/s1, Supplementary Method S1: Isolation and culture of peripheral blood cells (Protocol A); Supplementary Method S2: Isolation and culture of peripheral blood cells (Protocol C); Supplementary Method S3: Freezing, cryopreservation and thawing of peripheral blood cells isolated following Protocol C.
