# Supplementary material for: A β-Thalassemia Cell Biobank: Updates, Further Validation in Genetic and Therapeutic Research and Opportunities During (and After) the COVID-19 Pandemic
Source: J Clin Med. 2025 Jan 6;14(1):289. doi: 10.3390/jcm14010289 (PMC11722022; doi:10.3390/jcm14010289)
Supplement: Supplementary file 1 [file jcm-14-00289-s001.zip › jcm-3358731-supplementary.pdf]

Journal of
Clinical Medicine

MDPI

A "pre-COVID-19 β-Thalassemia Cell Biobank": updates, further validation in genetic and therapeutic research and opportunities during (and after) the COVID-19 pandemic

Roberto Gambari [1], Maria Rita Gamberini[2], Lucia Carmela Cosenza[2], Cristina Zuccato[2], and Alessia Finotti [2,*]

SUPPLEMENTARY MATERIALS

Supplementary Method 1: Isolation and culture of peripheral blood cells (Protocol A) (*)

Peripheral blood mononuclear cells (PBMCs) (from about 25 ml of blood) were collected in Vacutainer LH treated tubes (BD Vacutainers, Becton–Dickinson, UK). PBMCs isolation was obtained from whole blood by Ficoll-Hypaque density gradient centrifugation (Lympholyte®-H Cell Separation Media, Cedarlane, Euroclone, Italy). After the separation of the various blood components, the ring was harvested and washed once with 1× Dulbecco's Phosphate Buffered Saline without Ca & Mg (DPBS W/O CA-MG, GIBCO, Invitrogen, Life Technologies, Carlsbad, CA, USA). $CD34^+$ cells were selected from PBMCs using anti-$CD34^+$ magnetic microbeads and magnetic activated cells sorting separation LS columns (both from Miltenyi Biotec, Bergisch Gladbach, Germany) according to the manufacturer's protocol. The separation in the column was done twice to increase the $CD34^+$ cells purification. The obtained cells were maintained in culture with two different protocols. In the first protocol (Protocol A), the medium contained α-minimal essential medium (α-MEM, Sigma-Genosys, Saint Louis, Missouri, USA), prepared from a powder and diluted with water; a solution of Penicillin-Streptomycin (PEN-STREP 10,000 U/mL, Lonza, Verviers, Belgium); 10 % fetal bovine serum (FBS, Celbio, Milan, Italy); 10 % conditioned medium (CM), obtained from cell cultures of bladder cancer cells (5637); 1 μg/ml of cyclosporin A (Sigma-Aldrich, Saint Louis, Missouri, USA), prepared from cyclosporine absolute ethanol and diluted in 1X DPBS (GIBCO, Invitrogen, Life Technologies Carlsbad, CA, USA), in the ratio 1:1. After 7 days in Phase I culture, the non adherent cells were washed once with 1X DPBS (GIBCO, Invitrogen, Life Technologies Carlsbad, CA, USA), and then cultured in Phase II medium. This medium contains α-

MEM (Sigma Genosys, Cambridge, UK), 30 % FBS (Celbio, Milan, Italy), 1 % deionized bovine serum albumin (BSA, Sigma Genosys, Cambridge, UK), 10−5 M β-mercaptoethanol (Sigma Genosys, Cambridge, UK), 2 mM L-glutamine (Sigma Genosys, Cambridge, UK), 10−6 M dexamethasone (Sigma Genosys, Cambridge, UK), and 1 U/mL human recombinant erythropoietin (EPO Tebu-bio, Magenta, Milan, Italy), and stem cell factor (SCF, BioSource International, Camarillo, CA, USA) at the final concentration of 10 ng/mL.

Supplementary Method 2: Isolation and culture of peripheral blood cells (Protocol C) (*)

In Protocol C, the isolation of mononuclear cells was performed starting from 20–25 ml of peripheral blood collected before transfusion from patients who gave informed consent. A mixture of Blood and PBS 1× at a 1:1.5 ratio was stratified on top of Lympholyte®-H Cell Separation Media. A monolayer of PBMCs was obtained by centrifugation at 2000 rpm for 30 min, at room temperature, and carefully transferred into new tube. PBMCs were washed twice in 50 ml PBS 1×, and resuspended in 600 µl of BSA solution. The isolation of CD34+ cells from PBMCs was performed using the CD34+ MicroBead Kit, LS MiniMACS Column, by magnetic separation using an autoMACS Separator (all from Miltenyi Biotec). 100 µl of CD34+ MicroBead were added to the cell suspension and gently mixed for 15 min at 4 °C. Two washes were performed before proceeding with column separation. Cells were resuspended in 1 mL of beading buffer (PBS1X, 2mMEDTA, 0.5 %BSA) and loaded onto the column in two consecutive steps with beading buffer washes in between. To enrich for CD34+ cells, the eluted fraction was re-eluted into a second LS Column. Cells pellets were resuspended in 5 ml of growth medium. Five ml of expansion medium contains: StemSpan Serum-Free Medium Expansion (Voden, Vancouver, Canada), 50 µl of StemSpan® CC100 Cytokine Cocktail for Expansion of Human Hematopoietic Cells Stem Cell Technologies (Voden, Vancouver, Canada), 2 U/mL erythropoietin (EPO, Tebu-bio, Magenta, Milan, Italy), 10−6 M dexamethasone (Dexamethasone 21-phosphate disodium salt, Sigma-Aldrich, Saint Louis, Missouri, USA), 50 µl of a 100× Pennicilin/Streptomycin solution (Lonza, Verviers, Belgium). Cell growth and differentiation was monitored over time. Fresh expansion medium was added to

maintain cell confluence below 5 × 105 cells/mL. Cells were frozen between 7 and 12 days of expansion.

Supplementary Method 3: Freezing, cryopreservation and thawing of peripheral blood cells isolated following Protocol C (*)

Once the maximum in cell expansion was achieved, CD34+ cells were frozen in single vials of 5 × 106 cells each, following a previously described method [18] using a solution made of: 40 % Iscove's modified Dulbecco's Medium (IMDM, Life Tecnologies, Carlsbad, CA, USA), 50 % FBS (Celbio, Milan, Italy) and 10 % Dimethyl Sulfoxide RPE-ACS (DMSO, Carlo Erba, Italy). Cells were thawed by immediate incubation at 37 °C and resuspended dropwise in Iscove Modified Dulbecco's Medium (IMDM) with 5 % FBS. After a 10 min incubation at room temperature, the cell suspension was centrifuged at 1200 rpm at room temperature for 5 min, the supernatant removed and the cells suspended in expansion medium.

(*) These protocols/methods are modified from:

Reference

18 Cosenza et al., 2016 (Cosenza LC, Breda L, Breveglieri G, Zuccato C, Finotti A, Lampronti I, Borgatti M, Chiavilli F, Gamberini MR, Satta S, Manunza L, De Martis FR, Moi P, Rivella S, Gambari R, Bianchi N. A validated cellular biobank for β-thalassemia. J Transl Med. 2016 Sep 2;14(1):255. doi: 10.1186/s12967-016-1016-4). Copyrights can be found at: https://translational-medicine.biomedcentral.com/articles/10.1186/s12967-016-1016-4 (accessed on December 12, 2024). Further information can be found in the same article.
